# Adsorption of divalent cadmium by calcified iron-embedded carbon beads†

**DOI:** 10.1039/c9ra10309k

**Published:** 2020-02-10

**Authors:** Yalin Cheng, Kaiqian Wang, Biyang Tu, Shan Xue, Jiahui Deng, Haisheng Tao

**Affiliations:** Anhui Provincial Engineering Laboratory of Water and Soil Pollution Control and Remediation, School of Environmental Science and Engineering, Anhui Normal University Wuhu 241000 Anhui China taohaish@mail.ahnu.edu.cn; Anhui Laboratory of Molecule-Based Materials, School of Chemistry and Materials Science, Anhui Normal University Wuhu 241000 Anhui China

## Abstract

A novel iron-embedded carbon bead was prepared by the calcination of a calcium alginate gel bead mixed with iron nanoparticles coated by polydopamine. The prepared iron-embedded carbon bead was characterized by infrared spectrum analysis, X-ray diffraction, Raman spectroscopy, vibrating sample magnetometry, X-ray photoelectron spectroscopy, scanning electron microscopy and transmission electron microscopy. It was discovered that the novel structure efficaciously prevented the agglomeration of iron nanoparticles. Additionally, the effects of dose, pH, exposure time, temperature and initial concentration on the adsorption of Cd(ii) were studied, and the reusability of the material was analyzed. Fe/SA-C showed high Cd(ii) removal capability (220.3, 225.7, 240.8 mg g^−1^ at 288, 298, 308 K), easy recoverability and high stability. In addition, some slightly different interpretations of the adsorption mechanism are given. This study clearly revealed that Fe/SA-C has potential application in the removal of Cd(ii).

## Abstract 2

The material structure could prevent Fe nanoparticle agglomeration during synthesis and maintain Fe stability during adsorption. Fe/SA-C had a superior adsorption property and easy recoverability.

## Graphical abstract

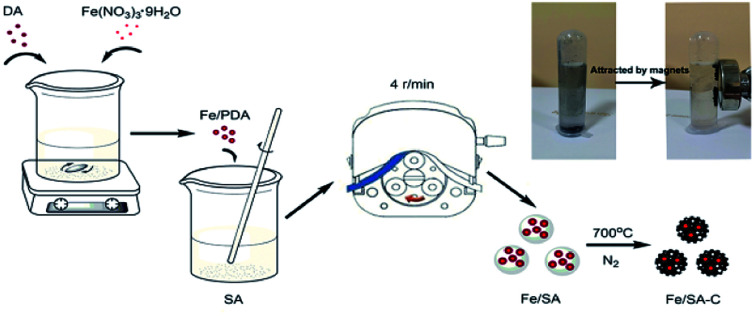

## 1 Introduction

Cadmium is considered to be one of the most common toxic metals in water, causing health problems and considerable environmental problems even in trace amounts because of its high toxicity, non-biodegradability and persistence.^1,2^ Therefore, numerous treatment techniques, such as chemical precipitation, ion exchange, reverse osmosis, nanofiltration, and adsorption are applied to mitigate cadmium pollution.^3^ Among these methods, adsorption is regarded as a promising technique and has been studied extensively for its economy, high efficiency, simplicity, and absence of secondary contamination compared with other techniques.^4^

Carbon is an attractive adsorption material with many advantages, such as low cost, easy processing, large specific surface area, excellent chemical stability and wide operating temperature range, and thus, numerous studies have reported the adsorption of pollutants by different carbon materials.^5–9^ However, a single carbon material cannot fully meet the needs of pollutant treatment because carbon material with uncontrollable properties is sometimes easily affected by adsorption conditions.^10^ So, loading appropriate metal particles or their oxides onto carbon material has attracted great attention; this method could increase the specific surface area and adsorption sites of the material. The metal-loaded carbon composite material not only has an excellent porous structure, thermodynamic stability and controllable chemical properties, but also a wider range of application than the single-carbon material.^11^ Aluminum, zirconium and other particles or their oxide particles are always considered to be appropriate materials for loading.^12–14^ For example, Al_2_O_3_/C composite, with an innovative and complicated preparation, was used to remove Pb(ii) and Cd(ii).^15^ Carbon nanotube-coated ZrO(OH)_2_ showed better adsorption properties than the uncoated material.^16^ The same distinction could be seen between the aggregates of manganese oxide impregnated graphene oxide and the unimpregnated graphene oxide.^17^ At the same time, the loading of magnetic nanoparticles has received considerable attention for its large specific surface area, high dispersion, superparamagnetism in water, and efficient separation from solution.^18^ The loaded manganese oxide or iron oxide material proved to have an excellent removal rate and easy recyclability.^19–21^ However, migration and surface agglomeration of metal nanoparticles might occur during loading, so it is necessary to wrap single-metal nanoparticles with inert material to prevent the agglomeration of metal particles.^22^ Carbon shell is a common coating material that not only stabilizes the internal metal particles, but also facilitates the solute diffusion, due to its mesoporous structure.^23^ In addition, adsorption materials are always made into beads, especially gel balls, to remove pollutants, which are easy to recycle but hard to store for their friability.^24,25^

In this work, iron nanoparticles were coated by polydopamine (PDA) and subsequently mixed in calcium alginate gel to prepare gel balls, which are then calcined into carbon beads. In this way, the iron-embedded carbon beads (Fe/SA-C) can efficaciously and simultaneously avoid the agglomeration of iron nanoparticles, facilitate recycling, and augment the stability of materials. The prepared material was characterized by Fourier transform infrared spectrum analysis (FTIR), X-ray diffraction (XRD), X-ray photoelectron spectroscopy (XPS), scanning electron microscope (SEM) and transmission electron microscope (TEM). The effects of dose, pH, exposure time, temperature and initial concentration on the adsorption of Cd(ii) were studied. The reusability of Fe/SA-C was analyzed, and the adsorption mechanism was discussed preliminarily through characterization, kinetic models, and thermodynamic models. Fe/SA-C showed high removal efficiency, easy recoverability and high stability. The adsorption kinetics and isotherms of Cd(ii) onto Fe/SA-C matched well with the pseudo-second-order kinetics model and the Langmuir model, respectively. Additionally, Cd(ii) adsorption onto Fe/SA-C was endothermic and spontaneous. The results reveal that Fe/SA-C has potential application for the removal of Cd(ii).

## 2 Materials and methods

### 2.1 Reagents

The chemicals used in the study included dopamine hydrochloride (McLean Biochemical Technology Co., Ltd.), calcium chloride (Xilong Chemical Co., Ltd.), sodium alginate, iron(iii) nitrate nonahydrate (Aladdin), anhydrous methanol, anhydrous ethanol, nitric acid (Shanghai Lingfeng Chemical Reagent Co., Ltd.), ammonia water and sodium hydroxide (Sinopharm Group Chemical Reagent Co., Ltd.). All reagents were of analytical grade, and no further purification was required prior to use. The water used in the study was ultrapure water (Millipore, ≥18.2 MΩ cm, 25 °C).

### 2.2 Preparation of iron-embedded carbon bead (Fe/SA-C)

In a typical synthesis of Fe encapsulated in polydopamine nanoparticle (Fe/PDA), 112 mL deionized water, 28 mL methanol, and 0.8 mL ammonia water were mixed for 30 min to obtain a uniform transparent solution and subsequently stirred for 30 h with 0.5 g dopamine hydrochloride (DA) and 0.88 mmol Fe(NO_3_)_3_·9H_2_O, and the reaction mechanism was similar to the silica-like Stöber process.^26,27^ The suspension was centrifuged, and the precipitate rinsed with deionized water and ethanol several times. The deposit was dried for 12 h at 60 °C. Finally, Fe/PDA particles, after grinding, were used for the next step.

The prepared Fe/PDA was mixed in 100 mL of 2% alginate hydrogel in the proportion of 1 : 1 with sodium alginate. Next, evenly mixed gel was dropped into a 4% CaCl_2_ solution to obtain hydrogel beads by peristaltic pump at a rate of 2.5 mL min^−1^. The obtained pellets were aged for 4 h, then washed and dried. Finally, the precursor was calcined at 700 °C for 2 hours under N_2_ atmosphere, and the obtained iron-embedded carbon beads (Fe/SA-C) were used for the study.

### 2.3 Characterization of iron-embedded carbon beads

The microstructure and the energy-dispersive X-ray spectroscopy of Fe/SA-C were characterized by scanning electron microscopy (S-8100, Zeiss, Germany) and transmission electron microscopy (Tecnai F20, FEI, USA). Composition and crystal structure of Fe/SA-C were obtained using a powder X-ray diffractometer (D8 Advance, Bruker, Germany), and the XRD spectrum was recorded in the range of 10–90°, with a step size of 0.02° and a step time of 0.04 s. Raman shift measurement was carried out *via* Raman spectrometer at 532 nm (Labram BX40, Horiba, France). The magnetic behavior was measured using a vibrating sample magnetometer at room temperature (7404, Lake Shore, USA). The chemical structure was analyzed by the Fourier transform infrared spectrometer (IR-Prestige-21, Shimadzu, Japan) in the wavenumber range of 400–4000 cm^−1^. XPS spectra of Fe/SA-C were recorded on a PHI 5000 series ESCA spectrometer (PerkinElmer).

### 2.4 Adsorption experiments

The effects of dosage, pH, and initial concentration were evaluated respectively by keeping the other factors in the optimal condition. 4, 8, 12, 16, 20, 24, 28, 32, 36, and 40 mg of Fe/SA-C were mixed in 50 mL of Cd solution with the concentration of 50 mg L^−1^ at the designated temperature (298 K) and pH (6), respectively. The experiment with pH was controlled by adjusting the pH in the range of 3–10 with 0.1 mol L^−1^ NaOH and HNO_3_ in 50 mL of 50 mg L^−1^ Cd(ii) solution, and the dose of Fe/SA-C was 8 mg. The influence of concentration of Cd(ii) was evaluated at different initial concentrations (20–180 mg L^−1^). Kinetic study was conducted, and the suspensions were sampled at regular intervals (2, 5, 10, 15, 30, 45, 60, 120, 240, and 480 min). Adsorption thermodynamics was examined at different temperatures of 288 K, 298 K and 308 K. Desorption experiment was carried out by adding the Cd-adsorbed Fe/SA-C into a dilute solution of HNO_3_ (10 vol%) for 30 min, and a regeneration experiment was repeatedly conducted 5 times on the recycled Fe/SA-C. All experiments were performed in 50 mL of 50 mg L^−1^ Cd solution with 12 mg Fe/SA-C at 298 K unless otherwise specified, and all treatments were repeated 3 times independently. All adsorption experiments were performed *via* a shaker incubator, and the agitation velocity was 150 rpm.

### 2.5 Analysis

When the adsorption reached equilibrium, the adsorption amount *q* and the removal rate *E* of Fe/SA-C for Cd(ii) in solution are described according to the following eqn (1) and (2):

1 *q* = (*C*_0_ − *C*_e_) × *V*/*m*

2 *E* = (*C*_0_ − *C*_e_)/*C*_0_ × 100%

where *C*_0_ and *C*_e_ are the initial and equilibrium concentrations of Cd(ii) in mg L^−1^; *V* is the volume of Cd(ii) solution in L; and *m* is the mass of Fe/SA-C in g.

The experimental results were fitted using the pseudo-first-order, pseudo-second-order and intra-particle diffusion models^28^ with the following equations, respectively:

3 ln(*Q*_e_ − *Q*_*t*_) = ln *Q*_e_ − *k*_1_ × *t*

4 *t*/*Q*_*t*_ = 1/(*k*_2_ × *Q*^2^_e_) + *t*/*Q*_e_

5 *Q*_*t*_ = *k*_3_ × *t*^1/2^ + *C*,

where: *k*_1_ (min^−1^), *k*_2_ (g (mg min)^−1^) and *k*_3_ (mg g^−1^ min^−1/2^) represent the adsorption rate coefficients of pseudo-first-order kinetics, pseudo-second-order kinetics and intra-particle diffusion equations, respectively; *Q*_*t*_ and *Q*_e_ are the adsorption capacity at time *t* and equilibrium in mg g^−1^; and *t* is the adsorption time in min.

Cd(ii) adsorption by Fe/SA-C was described by the Langmuir model (eqn (6)), the Freundlich model (eqn (7)) and the Temkin model (eqn (8)):^29,30^

6 *Q*_e_ = *Q*_m_ × *k*_L_ × *C*_e_/(1 + *k*_L_ × *C*_e_)

7 ln *Q*_e_ = ln *K* + (ln *C*_e_)/*n*

8 *Q*_e_ = *R* × *T* × *b*_t_ × ln(*a*_t_ × *C*_e_),

where: *Q*_e_ is the equilibrium adsorption capacity; *Q*_m_ is the single-layer saturated adsorption amount; *k*_L_ is the Langmuir adsorption characteristic constant in L g^−1^; and *C*_e_ is the mass concentration of Cd(ii) at equilibrium. *K* is a constant representing the adsorption capacity in mg g^−1^; *n* is a constant related to the adsorption strength and the surface heterogeneity of the adsorbent. *R* is the general gas constant; *T* is absolute temperature in K; *b*_t_ and *a*_t_ represent isotherm constants, respectively.

Further, thermodynamic calculations were carried out by the Van't Hoff equation:^31^

9 *K*_c_ = *Q*_e_/*C*_e_

10 Δ*G* = −*R* × *T* × ln *K*_c_

11 Δ*S* = (Δ*H* − Δ*G*)/*T*

In the equation, *Q*_e_ (mg g^−1^) and *C*_e_ (mg L^−1^) are the concentration of Cd(ii) in the adsorbent and solution, respectively; *K*_c_ is the equilibrium constant of adsorption; Δ*G* is the Gibbs standard free energy change variable in kJ mol^−1^; *R* is the gas constant; *T* is the thermodynamic temperature in K; Δ*H* is the standard enthalpy of adsorption in kJ mol^−1^; and Δ*S* is the standard entropy change of adsorption in J (mol K)^−1^.

## 3 Results and discussion

### 3.1 Characterization of SA-C

**Fig. 1:**
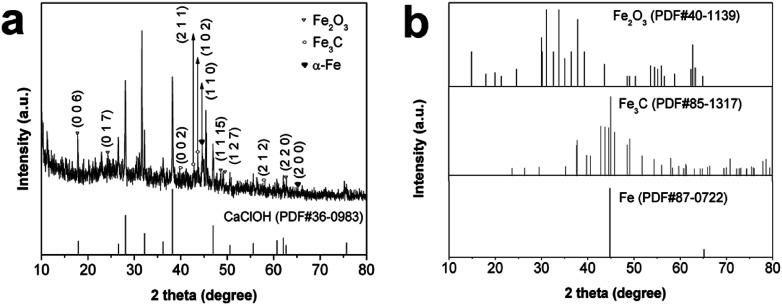
(a) XRD patterns of Fe/SA-C samples and CaClOH; (b) XRD standard patterns of Fe_2_O_3_, Fe_3_C, Fe.

XRD was used to analyze the crystal structure of Fe/SA-C, and the results are shown in Fig. 1. The diffraction peaks of Fe/SA-C at 44.8° and 65.2° corresponded to the (1 1 0) and (2 0 0) facets of α-Fe, indicating the presence of metallic Fe (PDF# 87-0722, Fig. 1b). Some diffraction peaks of Fe/SA-C were consistent with the characteristic diffraction of Fe_3_C, such as the peaks at 39.8°, 43.8°, 44.6° and 56.0°, which corresponded to the (0 0 2), (2 1 1), (1 0 2) and (2 1 2) facets of Fe_3_C (PDF# 85-1317, Fig. 1b). The results demonstrated that there was a small quantity of Fe_3_C existing in the Fe/SA-C. The formation of Fe_3_C gave the credit to the regional dissolution of carbon atoms into the Fe crystal lattice.^32^ A small portion of Fe_2_O_3_ from the chelation of Fe^3+^ with –OH groups during a pyrolysis process was also detected in XRD.^33^ The peaks at 14.9°, 24.6°, 49.1°, 50.4° and 62.8° could be well indexed to the (0 0 6), (0 1 7), (1 1 15), (1 2 7) and (2 2 0) characteristic diffractions of Fe_2_O_3_ (PDF# 40-1139). More remarkable, the sharp and well-defined reflection diffraction peaks of alkali-type calcium chloride could be found (PDF# 36-0983, Fig. 1a). The Raman spectrum of Fe/SA-C is shown in Fig. S1.† The D peak at 1356 cm^−1^ contributed to the defects (disordered carbon with sp^3^ bonding), and the G peak at 1602 cm^−1^ contributed to E_2g_ graphite mode (ordered graphitic structure with sp^2^ hybridization).^34^ The damage is estimated from the measurements of the disorder parameter given by *I*_D_/*I*_G_, where *I*_D_ and *I*_G_ are the integrated intensities of the D and G peaks, respectively. The *I*_D_/*I*_G_ is calculated to be 1.13.

**Fig. 2:**
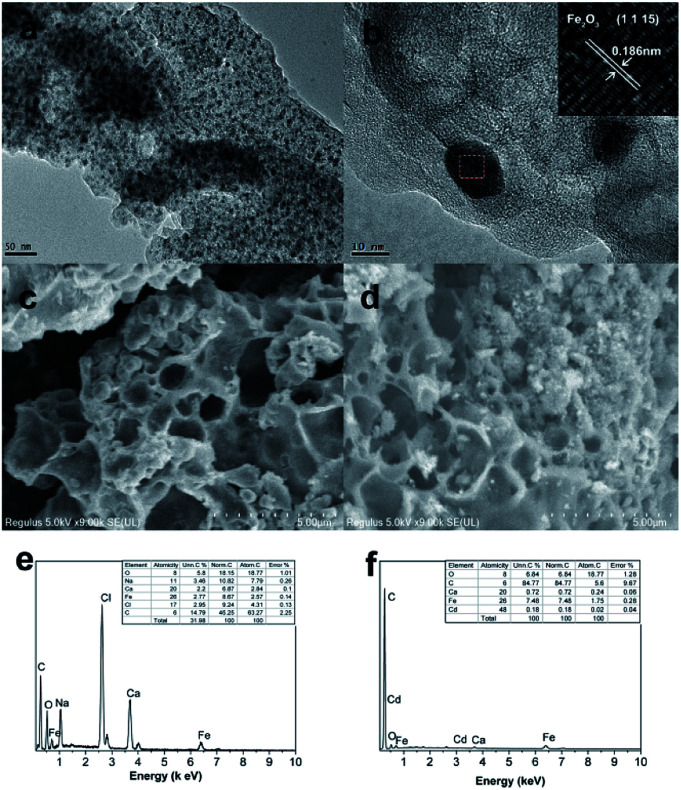
TEM images of (a and b) Fe/SA-C; SEM images of (c) as-prepared and (d) Cd-adsorbed Fe/SA-C; EDS of (e) as-prepared and (f) Cd-adsorbed Fe/SA-C.

The morphology and structure of Fe/SA-C were well manifested by TEM and SEM. It can be seen that the as-prepared Fe/SA-C is spherical, with a particle size of about 2 mm (Fig. S2†). In Fig. 2a and b, highly dispersive Fe_2_O_3_/Fe/Fe_3_C nanoparticles with a particle size of about 10 nm were embedded within the carbon entirely. In addition, atomic lattice fringes with spacing of 0.186 nm could be found, which corresponded to the interplanar spacing of {1 1 15} planes in Fe_2_O_3_ crystal, demonstrating that the Fe_2_O_3_/Fe/Fe_3_C nanoparticles were embedded into the carbon bead. Element analysis of Fe/SA-C is displayed in Fig. 2e, clearly showing the O peak located at 0.52 keV, C peak located at 0.24 keV, Cl peak located at 2.61 keV, Ca peaks located at 3.71 and 3.99 keV, and Fe peaks located at 0.71 and 6.40 keV. The SEM images of the as-prepared and Cd-adsorbed Fe/SA-C are shown in Fig. 2c and d, respectively. It could be seen that Fe/SA-C has a porous structure (Fig. 2c), while some pores were blocked after adsorption (Fig. 2d). In addition, the surface of the as-prepared Fe/SA-C was rougher than that of Cd-adsorbed Fe/SA-C, which may contribute to the adsorption of Cd(ii). This possibility was verified by EDS, where an additional obvious Cd peak at 3.13 keV was found in the post-adsorption characterization result (Fig. 2f).

**Fig. 3:**
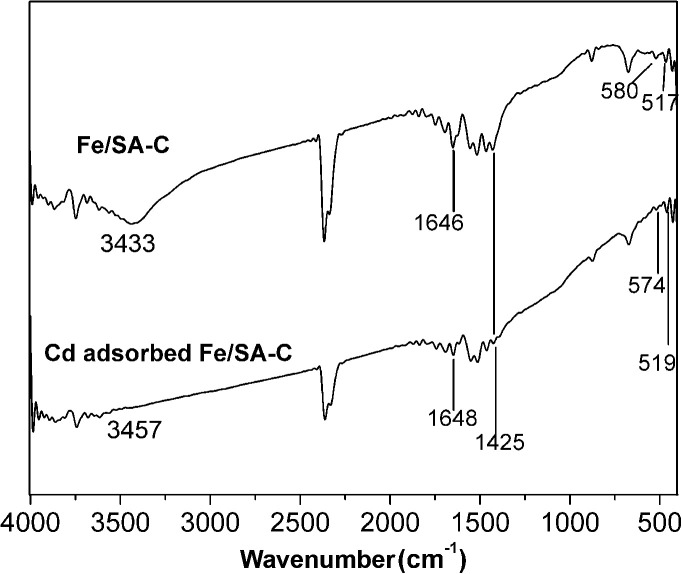
FTIR images of Fe/SA-C and Cd-adsorbed Fe/SA-C.

The representative FT-IR spectra of Fe/SA-C and Cd-adsorbed Fe/SA-C are shown in Fig. 3. In the spectrum of Fe/SA-C, it could be seen that a broad band at 3433 cm^−1^ is attributed to hydroxyl. The stretching vibration peak of C=O appeared at 1646 cm^−1^, and the peak at 1425 cm^−1^ might be assigned to the carboxylic group. In addition, peaks at 517 cm^−1^ and 580 cm^−1^ could be observed from the spectra of Fe/SA-C, which are characteristic of the stretching absorption mode of Fe–O.^35^ This evidence indicated that a few iron nanoparticles were successfully embedded into the carbon bead. For Cd-adsorbed Fe/SA-C, the hydroxyl peak at 3433 cm^−1^ of Fe/SA-C shifted to 3457 cm^−1^, and the intensity weakened, while blue shift of the Fe–O peak was observed, revealing that the hydroxyl group was mostly consumed during the adsorption of Cd(ii), and iron oxide nanoparticles might have been involved during the adsorption.

### 3.2 Adsorption performance studies

#### 3.2.1 Effect of Fe/SA-C dose

**Fig. 4:**
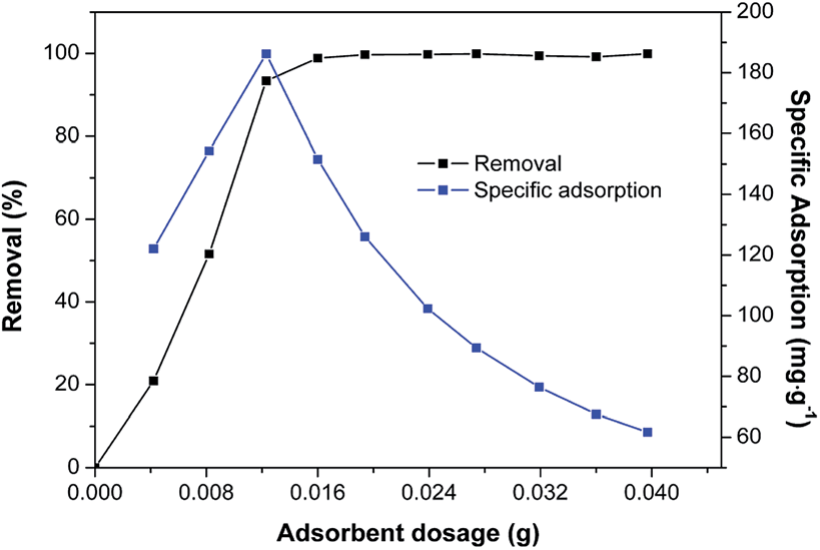
Effect of Fe/SA-C dosage on removal and specific adsorption. (*C*_0_ = 50 mg L^−1^, *T* = 298 K, pH = 6, and *t* = 240 min).

The effect of Fe/SA-C dose in solution on the removal rate and adsorption ratio of Cd(ii) was analyzed, and the result is shown in Fig. 4. The removal rate increased continuously with the increasing Fe/SA-C dosage, and the ultimate removal rate was more than 99.9% when the adsorbent dosage was 16 mg. Meanwhile, the change of specific adsorption went through two stages; the trend of adsorption ratio increased greatly in the first stage, contributing to the increase of effective adsorption sites, and the decline in the later stage was due to the saturated surface active sites.^20^ The maximum adsorption capacity was 186.2 mg g^−1^ when the dosage was 12 mg.

#### 3.2.2 Effect of solution pH

**Fig. 5:**
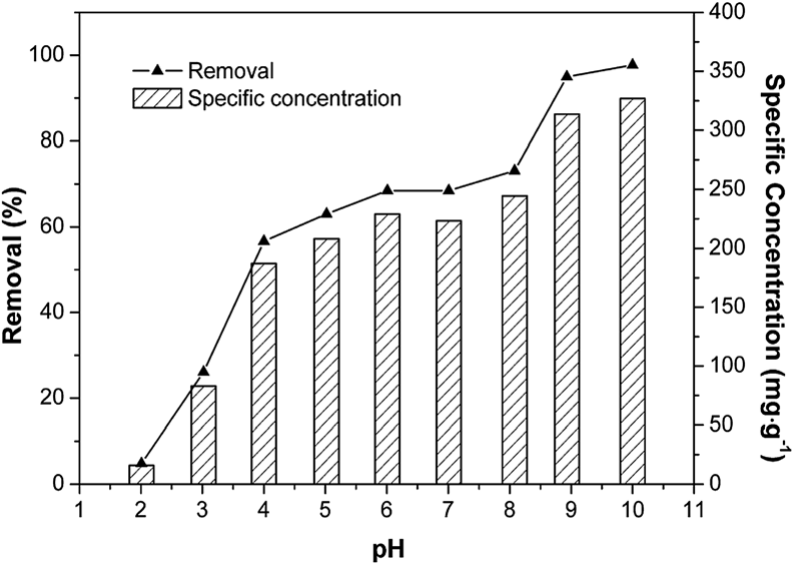
Effect of pH on removal and specific adsorption (*C*_0_ = 50 mg L^−1^, *T* = 298 K, *m*_adsorbent_ = 12 mg, and *t* = 240 min).

pH is a significant controlling factor of any aqueous solution-based adsorption process.^36^ Cd(ii) removal efficiency on Fe/SA-C was examined, and the results are displayed in Fig. 5. The Cd(ii) specific-adsorption increased markedly with the rise of solution pH from 2 to 4, whereas it remained stable at pH 4 to 8, and subsequently jumped from 8 to 10. Adsorption was inhibited when pH was less than 4, especially at pH = 2, for the reason that a large number of H^+^ ions competed for the adsorption sites on the material with Cd^2+^, resulting in a significant decline in the removal capacity.^37^ The adsorption rate and adsorption ratio of Fe/SA-C to Cd(ii) changed slightly, in which the adsorption ratio fluctuated around 200 mg g^−1^ when the pH was within the range of 4 to 8, and the peak of adsorption ratio was 222.6 mg g^−1^ at pH = 6. It was obvious that the adsorption process was less affected by the value of pH in this range. The removal rate of Cd(ii) jumped significantly when the pH of the solution was greater than 8, which might be due to the precipitation of Cd(ii).^38^ Therefore, the pH for Cd(ii) adsorption was optimized at 6 and applied in subsequent experiments.

#### 3.2.3 Kinetics analysis

**Fig. 6:**
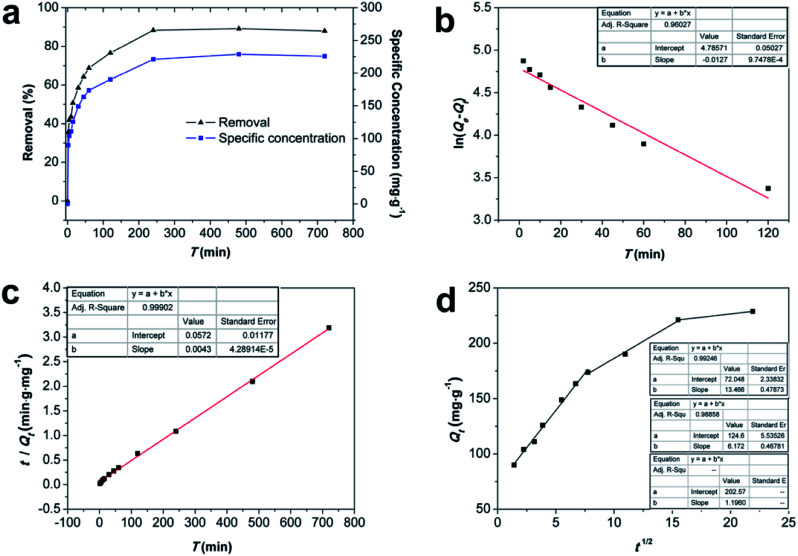
(a) Rates of adsorption of Cd(ii) by Fe/SA-C; (b) pseudo-first-order model; (c) pseudo-second-order model and (d) intraparticle diffusion model (*C*_0_ = 50 mg L^−1^, *T* = 298 K, *m*_adsorbent_ = 12 mg, and pH = 6).

The effect of contact time on the Cd(ii) adsorption performance was researched, and Fe/SA-C was studied by fitting the curves of the pseudo-first-order, pseudo-second-order, and intra-particle diffusion models (Fig. 6), and the kinetic fitting parameters are listed in Table 1. It is obvious from Fig. 6a that the adsorption capacity increased rapidly in the pre-60 min, which contributed to the large numbers of active sites on the surface of Fe/SA-C, and subsequently reached the adsorption equilibrium in four hours after a slow growth phase. The correlation coefficient (*R*^2^) of the pseudo-first-order kinetic was 0.960, and the calculated equilibrium adsorption capacity *Q*_e_ was not consistent with the actual adsorption capacity, while the *R*^2^ of the pseudo-second-order was 0.999, and the calculated *Q*_e_ was well accorded with the actual adsorption capacity, suggesting that the pseudo-second-order model was appropriate to describe the adsorption process. Thus, it can be concluded that the adsorption process of Cd(ii) on Fe/SA-C was mainly dominated by chemisorption, and it was mainly caused by the electron sharing or exchange between Cd(ii) and the functional group in Fe/SA-C.^39^

**Table 1:** Parameters obtained from different kinetic models

Model	Parameters		*R* ^2^
Pseudo-first-order	*k*_1_ (min^−1^)	*Q*_e_ (mg g^−1^)	
	0.013	119.786	0.960
Pseudo-second order	*k*_2_ (g (mg min)^−1^)	*Q*_e_ (mg g^−1^)	
	0.0003	230.947	0.999
Intra-particle diffusion	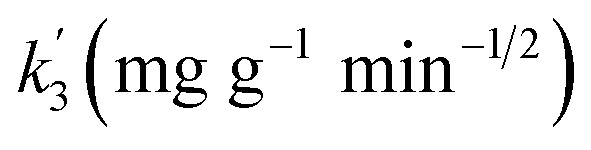	*C*′	
	13.466	72.049	0.992
	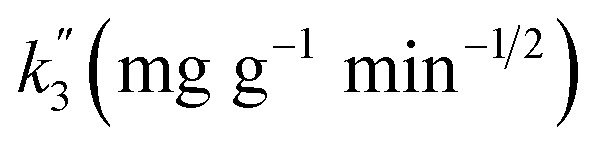	*C*′′	
	6.172	124.623	0.988
	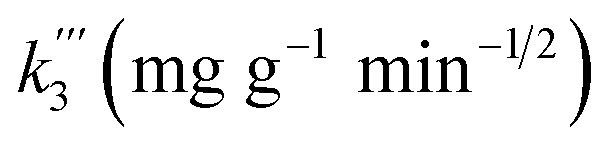	*C*′′	
	1.196	202.578	—

In addition, the fitted curve of the intraparticle diffusion model is shown in Fig. 6d. The fitted curve shows three segmented lines with different slopes, indicating that the whole adsorption process went through three different stages. Cd(ii) diffused to the surface of Fe/SA-C and occupied the active sites in the first stage, and then diffused to the pores slowly, and were subjected to internal diffusion. The third stage corresponded to the equilibrium period of reaction. There was no fitted curve passing through the origin, indicating that the factors controlling the reaction rate did not only include in-particle diffusion.^32^

#### 3.2.4 Adsorption thermodynamics

**Fig. 7:**
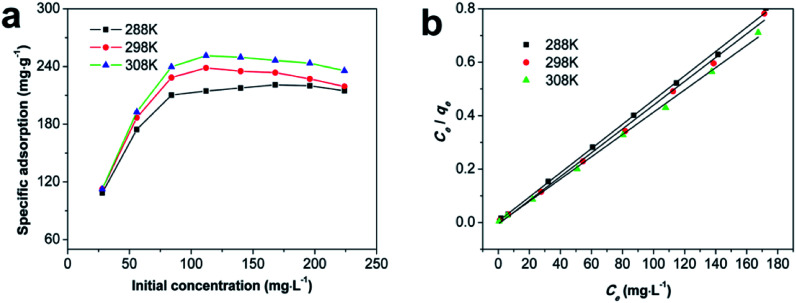
(a) Isotherm of Cd(ii) sorption onto Fe/SA-C at different temperatures; (b) linear fit of the Langmuir equation (*m*_adsorbent_ = 12 mg, *t* = 240 min and pH = 6).

The effect of initial concentration was examined at 288 K, 298 K, and 308 K, and the result is shown in Fig. 7. To better understand the mechanism of Cd(ii) adsorption onto Fe/SA-C, the Langmuir, Freundlich, and Temkin isotherm models and thermodynamic quantities were used to analyze Cd(ii) adsorption. It is clearly seen from Fig. 7 that the adsorption rate and the saturation adsorption amount on Fe/SA-C increased with the increment of temperature, indicating that the adsorption of Cd(ii) by Fe/SA-C was a spontaneous endothermic reaction, and the adsorption rate was accelerated by the increased temperature. It might be inferred that there was an increase in the diffusion of Cd(ii) toward the external surface and in the pores of Fe/SA-C, and a decrease in the metal transfer resistance with increasing temperature.^40^ The linear fits of the Langmuir, Freundlich and Temkin isotherm models are displayed in Fig. 7b and S3,† and the correlation parameters are given in Table 2. The Cd(ii) adsorption onto Fe/SA-C could be better described by the Langmuir model compared to the Freundlich model and Temkin model, because *R*^2^ values in the Langmuir model (*R*^2^ = 0.999, 0.997, 0.998 at 288 K, 298 K, 308 K, respectively) were greater than those of the Freundlich model (*R*^2^ = 0.626, 0.656, 0.726 at 288 K, 298 K, 308 K, respectively) and Temkin model (*R*^2^ = 0.662, 0.661, 0.733 at 288 K, 298 K, 308 K, respectively). It could be concluded that the Cd(ii) adsorption on Fe/SA-C was characterized by monolayer adsorption.^41^

**Table 2:** Parameters of isotherms for Cd(ii) sorption onto Fe/SA-C

*T*/K	Langmuir			Freundlich			Temkin		
	*Q*_m_	*k*_L_	*R* ^2^	*K*	*n*	*R* ^2^	*RT*/*b*_t_	*a*_t_	*R* ^2^
288	220.264	0.718	0.999	126.045	8.147	0.626	19.688	733.573	0.662
298	224.719	0.976	0.997	137.484	8.391	0.656	19.947	1374.228	0.661
308	239.808	1.020	0.998	144.402	8.132	0.726	21.372	1283.69	0.733

Thermodynamic parameters of Δ*H*, Δ*S*, and Δ*G* for Cd(ii) sorption onto Fe/SA-C were calculated from the van't Hoff equation and the plot between ln *K*_c_ and 1/*T*. The results are shown in Fig. S4† and Table 3. Δ*G* was the benchmark to determine whether a reaction was spontaneous, so the negative Δ*G* indicated that adsorption was spontaneous. The values of Δ*H* were positive, indicating that the adsorption process was endothermic. The values of Δ*S* were positive, suggesting the degree of freedom and randomness of adsorption process increased. For the spontaneous adsorption process, solvent molecules were replaced by adsorbate molecules, which caused the decrease of freedom and the increase of entropy.^42^

**Table 3:** Thermodynamic parameters for Cd(ii) removal onto Fe/SA-C

*T*	*K*_c_	Δ*G*	Δ*S*	Δ*H*
288	5.553	−4.105	128.027	23.516
298	5.959	−4.422		
308	6.189	−4.668		

Adsorption capacity of Cd(ii) by other adsorbents, along with that of the iron-embedded carbon bead, are listed in Table 4 for comparison. The adsorption capacities of different adsorbents were varied, and it is obvious that the maximum adsorption capacity of iron-embedded carbon bead was higher than most adsorbents.

**Table 4:** Comparison of adsorption capacity of Cd(ii) on various materials

Materials	Adsorption capacity (mg g^−1^)	pH	Temperature (K)	Ref.
Struvite attapulgite (MAP/APT)	121.14	5.45	298	3
Palm oil sludge biochar (POSB)	46.2	5.5	298	36
Fe-montmorillonite	25.7	5	298	43
Ferromanganese binary oxide–BC composites (FMBC)	101.0	6	308	44
Alginate–chitosan hybrid gel beads	6.63	3.5	298	45
Phosphate-embedded calcium alginate beads	82.64	5.5	298	46
Iron-embedded carbon beads	222.6	6	298	Present study

#### 3.2.5 Magnetism and reusability

Desorption experiments were also carried out in 50 mg L^−1^ of Cd(ii). The Cd(ii) on Fe/SA-C was eluted with HNO_3_ and separated by magnetic force. The collected Fe/SA-C was again subjected to the adsorption experiment at the optimum condition. Fig. S5† shows the adsorption capacity change curve when the cycle was repeated 5 times. It is obvious that the adsorption decreased significantly in the first two elutions, then gradually reached equilibrium; the final adsorption capacity was about 40 mg g^−1^, which might be due to the destruction of the material by pickling. Fortunately, the adsorbent remained intact after four regenerations and could be still applied several times.

Fig. S6a† displays the ferromagnetic behavior of Fe/SA-C. The saturation magnetization (*M*_s_) of Fe/SA-C is 5.98 emu g^−1^, the residual magnetic force (*M*_r_) is 1.26 emu g^−1^, and the coercivity (*H*_c_) is 592 *O*_e_. In addition, it is obvious from Fig. S6b† that Fe/SA-C, evenly dispersed in water, was accumulated under the attraction of a magnet. Therefore, it can be concluded that the Fe/SA-C can be recycled easily.

### 3.3 Sorption mechanism

The sorption mechanism of Cd(ii) on Fe/SA-C was demonstrated by FTIR, EDS and XPS. As shown in Fig. 3, the functional groups change significantly before and after adsorption, especially hydroxyl and carboxyl. For O–H, on the one hand, it was mostly consumed in the process of the adsorption of Cd(ii) to form strong mono- or multidentate inner-sphere complexes (*e.g.*, COO–Cd and Fe–O–Cd), leading to a shift of the characteristic O–H band;^43^ on the other hand, it participated in the precipitation to form the deposits of Cd(OH)_2_ in the material surface, which could explain the change trend seen in the pH influence experiment. When pH was too acidic, the combination of H^+^ and –OH inhibited the precipitation, resulting in the decrease of the adsorption capacity. The presence of carboxyl promoted the formation of hydrogels on the surface of Fe/SA-C. α-l-Guluronic acid (G) of alginate unchanged significantly after calcination, and the –COO^−^ connected to G was converted into –COOH under acidic conditions, which resulted in the decrease of hydrophilicity and the contraction of molecular chains. When pH increased, –COOH dissociated, and the hydrophilicity of the material increased; ion exchange occurred between Ca^2+^ in G and Cd^2+^, and G accumulated to form a cross-linked network structure, thus forming hydrogel, which fixed the Cd(ii) in the solution.^47^ This also explains the increase of Cd content in the EDS spectrum after adsorption (Fig. 2f).

**Fig. 8:**
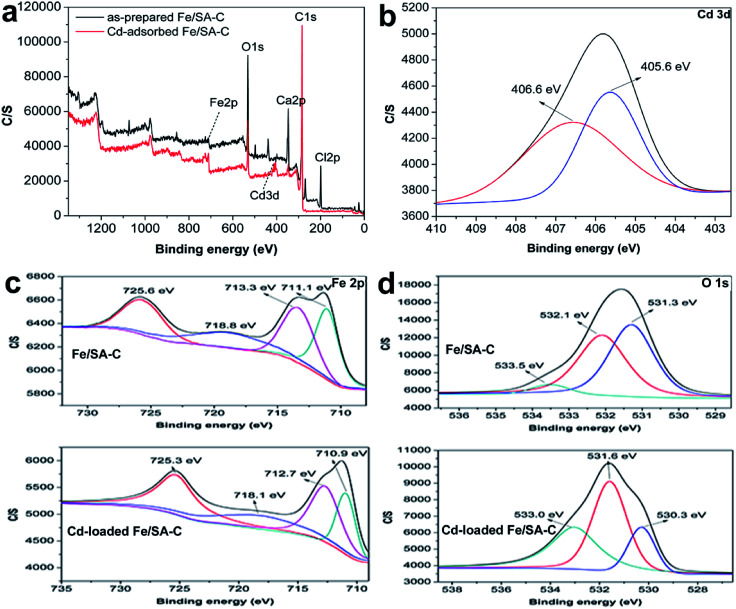
(a) XPS spectra of Fe/SA-C before and after adsorption of Cd(ii), (b) Cd 3d spectra of Fe/SA-C after adsorption, (c) Fe 2p spectra of Fe/SA-C before and after adsorption, (d) O 1s XPS spectra of Fe/SA-C before and after adsorption.

XPS analyses of the as-prepared and Cd-adsorbed Fe/SA-C are shown in Fig. 8. The total scans of XPS spectra showed that Fe/SA-C contained C, O, Cl, Ca and a small amount of Fe element, while the additional characteristic peak of Cd 3d in the Fe/SA-C presented after adsorbing Cd(ii) (Fig. 8a). The result demonstrates that Cd(ii) is adsorbed by Fe/SA-C. For the spectrum shown in Fig. 8c, the binding energies of 711.1 and 713.3 eV can be ascribed to Fe(iii) species, such as Fe_2_O_3_. It had no change after adsorption. However, the core-level binding energies of Fe decreased after adsorption of Cd(ii), which indicated that the negative charge of Fe increased, and Fe also participated in the adsorption as a Lewis acid. In addition, a decrease of Fe 2p spectra intensity was observed after adsorption, indicating the occurrence of interactions between Cd(ii) and Fe.

The three peaks in the O 1s spectra at 531.3, 532.1, and 533.5 eV (Fig. 8c) suggest the existence of various oxygen-containing compounds, such as organic oxygen and inorganic oxygen. In the O 1s region, the peaks at 531.3 and 532.1 eV can be ascribed to strongly bound inorganic oxygen in Fe_2_O_3_, and the peak at 533.5 eV can be attributed to C–O/C–OH. A decrease in the core-level binding energies of O after Cd(ii) adsorption indicates that O also participates in the adsorption as an electron-gaining Lewis acid.

As shown in Fig. 8b, the Cd(ii) peaks located at 406.6 and 405.6 eV, which basically correspond to the peak value for Cd_3_d_5_ in the literature,^44^ indicate that the chemical valence of Cd does not change during the adsorption process and no redox reaction occurs; that is, Cd(ii) on the surface of Fe/SA-C is present in the form of Cd(OH)_2_ and CdO. Therefore, it can be concluded that the deposition and chelation of Cd(ii) plays a significant role in the adsorption process.

## 4 Conclusions

In summary, iron-embedded carbon bead (Fe/SA-C) was synthesized successfully by high-temperature reduction. It was clear that the Fe/SA-C with well-dispersed Fe nanocrystals was easily prepared and separated from solution. The adsorption experiments showed that Cd(ii) removal by Fe/SA-C was less affected by pH, and the Cd(ii) adsorption kinetics and isotherms onto Fe/SA-C matched well with the pseudo-second-order kinetic model and the Langmuir model, respectively. Additionally, Cd(ii) adsorption onto Fe/SA-C was endothermic and spontaneous. Moreover, Cd(ii) removal by Fe/SA-C can be repeated multiple times. The results indicate that the proposed Fe/SA-C provides a novel low-cost candidate to treat sewage containing Cd(ii).

## Conflicts of interest

There are no conflicts to declare.

## Supplementary Material

RA-010-C9RA10309K-s001
