# Supplementary material for: Adsorption of divalent cadmium by calcified iron-embedded carbon beads
Source: RSC Adv. 2020 Feb 10;10(11):6277–86. doi: 10.1039/c9ra10309k (PMC9049697; doi:10.1039/c9ra10309k)
Supplement: RA-010-C9RA10309K-s001 [file RA-010-C9RA10309K-s001.pdf]

Electronic Supplementary Material (ESI) for RSC Advances.
This journal is © The Royal Society of Chemistry 2020

Table of contents

1. Figure S1. Raman spectra of Fe/SA-C.
2. Figure S2. SEM image of Fe/SA-C bead.
3. Figure S3. Linear fit of (a) Freundlich model and (b) Temkin model at different temperatures.
4. Figure S4. The plot between lnK_c and $1/T$.
5. Figure S5. Reuse efficiency of adsorbent.
6. Figure S6. (a) Magnetic hysteresis curve of Fe/SA-C at room temperature; (b) magnetic propriety of Fe/SA-C.

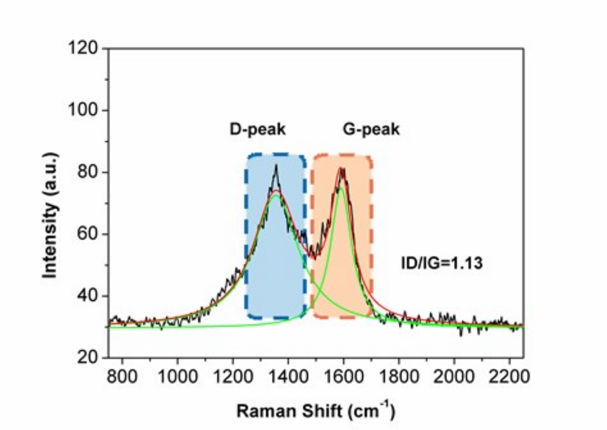

Figure S1. Raman spectra of Fe/SA-C.

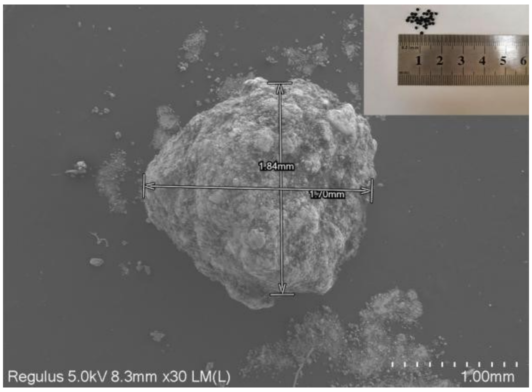

Figure S2. SEM image of Fe/SA-C bead.

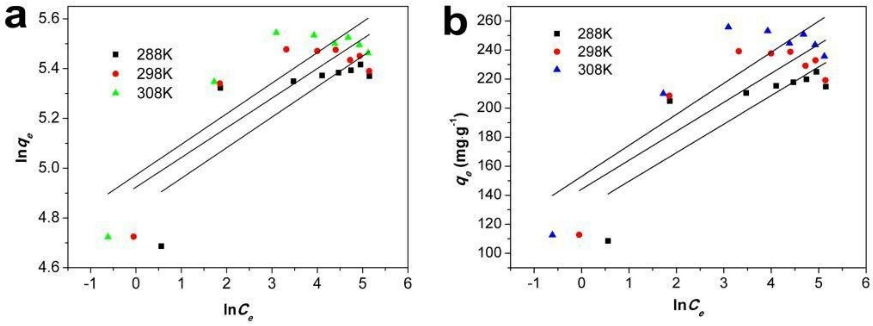

Figure S3. Linear fit of (a) Freundlich model and (b) Temkin model at different temperatures.

Linear equations at different temperatures are listed below:

Freundlich model:

288K: $\ln q_e=0.1227\ln C_e+4.8366$ ($R^2=0.626$)

298K: $\ln q_e=0.1191\ln C_e+4.9235$ ($R^2=0.656$)

308K: $\ln q_e$=0.1229$\ln C_e$+4.9726 (R^2=0.726)
Temkin model:
288K: q_e=19.688$\ln C_e$+129.90 (R^2=0.662)
298K: q_e=19.947$\ln C_e$+144.13 (R^2=0.661)
308K: q_e=21.372$\ln C_e$+152.97 (R^2=0.733)

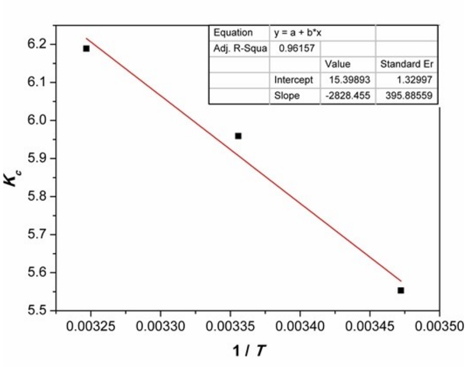

Figure S4. The plot between $\ln K_c$ and $1/T$.

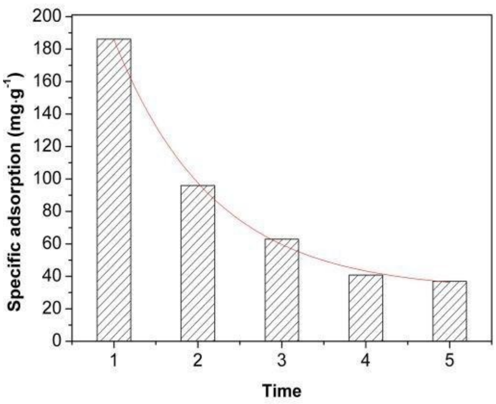

Figure S5. Reuse efficiency of the adsorbent.

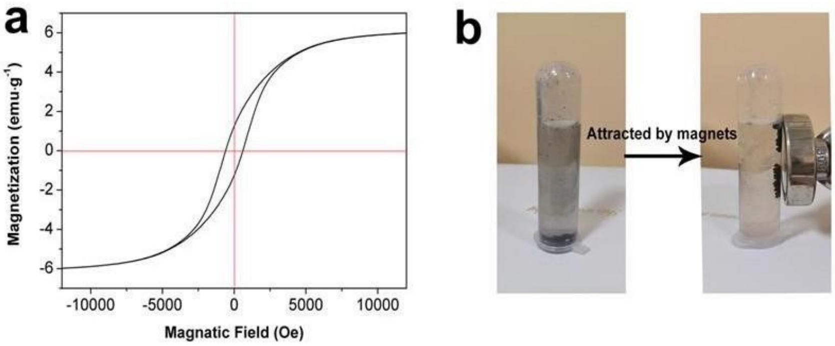

Figure S6. (a) Magnetic hysteresis curve of Fe/SA-C at room temperature; (b) magnetic propriety of Fe/SA-C.
